# 晚期非小细胞肺癌靶向治疗进展与展望：聚焦小分子酪氨酸激酶抑制剂

**DOI:** 10.3779/j.issn.1009-3419.2017.04.09

**Published:** 2017-04-20

**Authors:** 国伟 张

**Affiliations:** 450003 郑州，郑州大学附属肿瘤医院（河南省肿瘤医院）内科 Department of Internal Medicine, the Affiliated Cancer Hospital of Zhengzhou University, Henan Cancer Hospital, Zhengzhou 450003, China

**Keywords:** 肺肿瘤, 靶向治疗, 生物标记, EGFR, ALK, Lung neoplasms, Molecular targeted therapy, Biomarker, EGFR, ALK

## Abstract

目前晚期非小细胞肺癌的治疗已经迈入靶向时代并且发展迅速，药物不断推陈出新。小分子酪氨酸激酶抑制剂占据了其中最大的一块版图，它们往往有明确的分子靶标作为疗效预测因素，在特定分子分型的患者中表现出卓越的疗效，因此成为靶向治疗的典型代表。表皮生长因子酪氨酸激酶抑制剂厄洛替尼、吉非替尼、埃克替尼和间变性淋巴瘤激酶酪氨酸激酶抑制剂克唑替尼带来了里程碑式的进步。而近年来新一代酪氨酸激酶抑制剂在上述两类药物获得性耐药患者中又取得了巨大的成功，同时新的治疗靶点也不断涌现。本文就此对重要的药物和临床研究进行了梳理和总结，并对未来的发展做出展望。

## Abstract (en)

At present the treatment of advanced non-small cell lung cancer enters a targeted era and develops rapidly. New drugs appear constantly. Small molecular tyrosine kinase inhibitors have occupied the biggest piece of the territory, which commonly have a clear biomarker as predictor, and show remarkable effect in specific molecular classification of patients. The epidermal growth factor tyrosine kinase inhibitors such as gefitinib, erlotinib, icotinib and anaplastic lymphoma kinase tyrosine kinase inhibitors crizotinib have brought a milestone advance. In recent years new generations of tyrosine kinase inhibitors have achieved a great success in patients with acquired resistance to the above two kinds of drugs. At the same time new therapeutic targets are constantly emerging. So in this paper, we reviewed and summarized the important drugs and clinical trails on this topic, and made a prospect of the future development.

肺癌是全球癌症相关死亡的首要原因^[1]^，而非小细胞肺癌（non-small cell lung cancer, NSCLC）约占肺癌的85%^[2]^。晚期NSCLC的传统治疗模式是含铂双联化疗，中位总生存期（overall survival, OS）约8个月，2年生存率12%^[3]^。近年来晚期NSCLC的治疗快速发展，而关键性的进步来自于表皮生长因子受体酪氨酸激酶抑制剂（epidermal growth factor receptor tyrosine kinase inhibitors, EGFR-TKIs）^[4]^和间变性淋巴瘤激酶酪氨酸激酶抑制剂（anaplastic lymphoma kinase tyrosine kinase inhibitor, ALK-TKI）^[5]^。小分子酪氨酸激酶抑制剂代表着一种将NSCLC根据驱动基因区分为不同的分子亚型并给予相应药物的治疗模式，是晚期NSCLC靶向治疗的典型代表，也可称其为狭义的靶向治疗（广义的靶向治疗还包括抗血管生成治疗、免疫检查点抑制剂等）。随着EGFR-TKIs和ALK-TKI临床中的应用日趋成熟，小分子TKI类药物的发展开始有了新的主题和侧重，即解决EGFR-TKIs和ALK-TKI耐药后的治疗困境，并拓展更多的治疗靶点（本文提及的靶点及对应靶向药物的疗效总结见表 1）。本文拟就此进行分析和阐述。

**1:** 本文中提及的靶点及对应的靶向药物 Targets and corresponding drugs mentioned in this article

Targets	Drugs	Patients	ORR	Median PFS(mo)
EGFR	Osimertinib	Acquired resistance to first-generation EGFR-TKls	51%^[12]^	8.2^[12]^
		Acquired resistance to first-generation EGFR-TKls due to T790M mutation	58%-71%^[12-15]^	9.6-10.1^[12, 15]^
		TKI-naive classic *EGFR* mutations and/or T790M mutation	77%^[21]^	19.3^[21]^
	Rocliletinib	Acquired resistance to first-generation EGFR-TKls due to T790M mutation	21.8%-31.4%^[17]^	NR
	Olmutinib	Acquired resistance to first-generation EGFR-TKls due to T790M mutation	56%^[19]^	7.0^[19]^
ALK	Ceritinib	ALK+ patients who were crizotinib-resistant	38.6%-56%^[25, 26]^	5.7-8.3^[25, 26]^
		ALK+ patients who were crizotinib-naive	72.5%^[33]^	16.6^[33]^
	Alectinib	ALK+ patients who were crizotinib-resistant	48%-50%^[28, 29]^	8.1-8.9^[28, 29]^
		ALK+ patients who were crizotinib-naive	93.5%^[27]^	> 20.3^[32]^
	Brigatinib	ALK+ patients who were crizotinib-resistant	46%-72%^[30, 31]^	8.8-11.1^[31]^
	Lorlatinib	ALK+ or ROS1+ (mostly TKI-resistant)	50%^[31]^	NR
KRAS	Selumetinib+Docetaxol	*KRAS* mutation	37%^[42]^	5.3^[42]^
ROS1	Crizotinib	*ROS1* rearrangement	69%-72%^[47, 48]^	19.2^[47]^
MET	Crizotinib	MET amplification	33%^[52]^	NR
		MET exon 14 skipping	67%^[55]^	NR
	Capmatinib	MET overexpression	29%^[53]^	NR
		MET amplification	63%^[53]^	NR
RET	Cabozantinib	*RET* rearrangement	28%^[57]^	7^[57]^
	Vandetanib	*RET* rearrangement	17%-53%^[58, 59]^	4.7^[58]^
BRAF	Vemurafenib	*BRAF* V600E mutation	42%^[62]^	7.3^[62]^
	Dabrafenib	*BRAF* V600E mutation	33%^[63]^	5.5^[63]^
	Dabrafenib+Trametinib	*BRAF* V600E mutation	63%^[64]^	9.7^[64]^
HER2	TKIs like afatinib, *etc*/ Trastuzumab+Chemotherapy	*HER2* exon 20 mutation	50%^[66]^	5.1^[66]^
EGFR: epidermal growth factor receptor; ALK: anaplastic lymphoma kinase; EGFR-TKIs: EGFR tyrosine kinase inhibitors; ORR: objective response rate; PFS: progression free survival.				

## 1 第三代EGFR-TKIs

尽管*EGFR*敏感突变型NSCLC患者第一代EGFR-TKIs疗效卓越，但通常在9个月-14个月就会出现疾病进展^[4, 6, 7]^。一代EGFR-TKIs耐药机制多种多样，包括*EGFR*的二次突变、旁路途径的激活、以及病理亚型的转换等，其中尤以*EGFR* 20外显子T790M突变最为常见，可见于50%以上的获得性耐药患者^[8]^。既往耐药后除了传统化疗之外缺乏有效的治疗手段，虽然有增加药物剂量、换用另一种TKI等探索，但结果差强人意。第二代EGFR-TKIs为不可逆的泛HER抑制剂（阿法替尼、Dacomitinib等），除EGFR外尚可拮抗其他HER家族受体，并在基础研究中显示出一定的抗T790M活性，曾被寄望治疗一代TKIs获得性耐药患者，但临床试验给出了阴性的结果^[9]^。2009年问世的一种化合物WZ4002，在基础研究中拮抗T790M的活性30倍-100倍于一代TKIs，而对野生型EGFR的活性则约为一代TKIs的1/100^[10]^。此后这一类药物被称为第三代EGFR-TKIs，共同特征是对传统敏感突变以及T790M突变高度敏感，而几乎不抑制野生型*EGFR*。WZ4002未进行后续的临床研发。目前最主要的第三代EGFR-TKIs包括Osimertinib（AZD9291）、Rociletinib（CO-1686）、Olmutinib（HM61713）等。

### 1.1 Osimertinib（AZD9291）

Osimertinib是第一个获批的三代EGFR-TKIs^[11]^，也是目前为止第一个获得了Ⅲ期临床试验数据的三代EGFR-TKIs。其获批数据主要来自于Ⅰ期/Ⅱ期临床研究AURA及Ⅱ期临床研究AURA2。

AURA研究Ⅰ期阶段共入组253例一代TKIs获得性耐药患者。所有可评价疗效患者的客观缓解率（objective response rate, ORR）为51%，疾病控制率（disease control rate, DCR）为84%，扩展队列的无进展生存期（progression free survival, PFS）为8.2个月。其中127例T790M突变患者中，ORR为61%，DCR为95%，中位PFS为9.6个月^[12]^。AURA研究Ⅱ期阶段选择80 mg的治疗剂量，所入组人群为T790M突变的TKI获得性耐药者，2015年世界肺癌大会（World Conference on Lung Cancer, WCLC）报告其初步结果：入组患者201例，中位治疗暴露时间4.9个月，168例患者仍在治疗中，独立评价委员会评价ORR为58%，DCR为92%，研究者评估ORR为68%，中位PFS尚未达到^[13]^。AURA2是一项单臂的Ⅱ期临床研究，也入组T790M突变的耐药患者，入组患者210例，中位治疗暴露时间4个月，截至报告183例患者仍在治疗中，独立评价委员会评价ORR为64%，DCR为90%，研究者评价ORR为64%，中位PFS尚未达到^[14]^。

AURA 3是一项在一线EGFR-TKIs进展后且T790M突变阳性的患者中对比Osimertinib和培美曲塞含铂化疗疗效的Ⅲ期临床试验。结果显示与化疗相比，Osimertinib显著延长PFS（10.1个月*vs* 4.4个月；HR=0.3；95%CI：0.23-0.41；*P* < 0.001），ORR也显著优于化疗：71% *vs* 31%（OR=5.39, 95%CI: 3.47-8.48, *P* < 0.001）。该研究确立了Osimertinib在一线TKIs进展后T790M突变阳性患者中的首选地位^[15]^。

### 1.2 Rociletinib（CO1686）

Rociletinib是另一种广受关注的第三代EGFR-TKIs。目前主要的临床数据来自于Ⅰ期/Ⅱ期临床研究TIGER-X，该研究分为2个阶段，Ⅰ期的剂量爬坡阶段和Ⅱ期的剂量扩展阶段。研究最初入组的57例患者应用游离型Rociletinib，之后入组的患者应用氢溴酸结合型1686（药代动力学更优）。在Ⅱ期扩展阶段中，纳入T790M阳性的患者接受500 mg每日2次、625 mg每日2次、或750 mg每日2次的治疗剂量。截至2015年文献发表，共入组130例患者，客观缓解见于游离型900 mg每日2次剂量组及氢溴酸盐所有剂量组，在这些患者中，T790M阳性者ORR为59%，DCR为93%；T790M阴性者ORR为29%，DCR为59%。T790M阳性和阴性患者的中位PFS分别为13.1个月和5.6个月。在研究中Rociletinib显示出两种特征性的不良反应：高血糖症，发生率高达47%，2级以上发生率为22%；QT波延长，发生率为12%，3级以上为5%；但未有治疗相关性死亡^[16]^。

基于TIGER-X研究的结果，同Osimertinib一样，2015年Rociletinib进入了食品药品监督管理局（Food and Drug Administration, FDA）的快速审批通道。但由于FDA认为TIGER-X研究中患者的疗效评价未按照实体瘤疗效评价标准（Response Evaluation Criteria in Solid Tumors, RECIST）评价标准进行严格的确认，并且对于高血糖和QT波延长的不良反应存在疑虑，最终于2016年4月拒绝提前批准Rociletinib。随后2016年美国临床肿瘤学会（American Society of Clinical Oncology, ASCO）年会中报道了TIGER-X研究的数据更新，并对疗效进行了确认，确认后T790M阳性患者中500 mg每日2次剂量组的ORR仅21.8%，625 mg每日2次剂量组的ORR仅31.4%^[17]^。

### 1.3 Olmutinib（HM61713）

Olmutinib（HM61713）是一种韩国研发的第三代EGFR-TKIs。2015年美国临床肿瘤学会（American Society of Clinical Oncology, ASCO）年会报告了其Ⅰ期/Ⅱ期临床试验两个扩展队列的初步结果：300 mg队列（*n*=83）和800 mg队列（*n*=62，Ⅱ期阶段），800 mg队列中所有患者均存在T790M突变，为Ⅱ期临床研究推荐剂量。结果显示两队列的ORR分别为29.1%和54.8%^[18]^。2016年ASCO年会报告了Ⅱ期阶段（800 mg）的数据更新：共入组76例T790M阳性患者，至数据截止，中位治疗持续时间为7个月，34%的患者仍在接受治疗。ORR为56%（经过确认的ORR为44%），DCR为90%，中位PFS 7.0个月^[19]^。2016年5月Olmutinib在韩国被批准用于治疗T790M阳性的进展期NSCLC，成为全球第二个获批的第三代EGFR-TKIs^[20]^。

其他的第三代EGFR-TKIs还包括EGF816、ASP8273以及我国原研的Avitinib等，多已初步展现了良好的临床疗效。总之，第三代EGFR-TKIs对于一代TKIs获得性耐药患者的作用已毋庸置疑，尤其在T790M突变患者中，有几乎等同于一线TKIs在敏感人群中的疗效。不同药物之间，从目前数据看来Osimertinib疗效最优，但仍需有进一步证据支持。而第三代EGFR-TKIs也已经有了初步的一线治疗数据：2016年欧洲肺癌大会（European Lung Cancer Conference, ELCC）报告AURA Ⅰ期扩大研究中Osimertinib一线治疗队列的结果：入组60例患者（80 mg, *n*=30; 160 mg, *n*=30），40% *EGFR* 19外显子突变，42% *EGFR* L858R突变，5例存在*EGFR* T790M突变。ORR为77%（其中80 mg组67%，160 mg组87%），DCR为98%，中位PFS更是达到惊人的19.3个月^[21]^。因此将来第三代TKIs究竟是作为一代、二代TKIs耐药后的解救治疗，还是直接走向一线取代传统TKIs，尚未可知。

## 2 新一代ALK-TKIs

与EGFR-TKIs相似，大部分ALK阳性的NSCLC患者在接受克唑替尼治疗1年内就会出现进展^[5, 22]^。克唑替尼获得性耐药的机制大体上可以被区分为两类^[22]^。第一类可称之为ALK靶点依赖性耐药：因为这些机制的出现增强、恢复了ALK通路的激活，它主要包括ALK激酶域的继发性突变和*ALK*融合基因的扩增，两者可单独或同时出现，尤以继发性突变最为常见，见于约30%的耐药患者；另一类为非ALK靶点依赖性耐药，即旁路激活：如*EGFR*、*KRAS*获得性突变，*c-KIT*基因扩增等。所谓新一代ALK-TKIs，对EML4-ALK的抑制能力强于克唑替尼，且对导致克唑替尼耐药的继发性突变多有不同程度的抑制能力（不同药物对不同突变型的抑制谱不同）。因此，新一代ALK-TKIs可以对抗第一类机制导致的耐药，有望成为克唑替尼耐药后的主要治疗手段。

### 2.1 已经获批的新一代ALK-TKIs

目前全球获批的新一代ALK-TKIs包括Ceritinib和Alectinib。

基于2014年Ⅰ期临床试验ASCEND-1报告的初步临床结果，Ceritinib获得FDA批准用于克唑替尼耐药的晚期ALK阳性NSCLC患者^[23]^。当时在克唑替尼耐药及未经ALK-TKIs治疗的ALK阳性患者中，ORR分别为55%和66%，整体PFS为8.2个月，耐药患者为6.9个月^[24]^。在2016年更新后的研究结果中，共83例未经ALK-TKI治疗及163例曾经ALK-TKI治疗的ALK阳性患者，ORR分别为72%和56%，中位PFS分别为17.0个月和8.3个月^[25]^。而在另一项评估Ceritinib治疗克唑替尼耐药后患者疗效的Ⅱ期临床试验ASCEND-2^[26]^中，入组140例患者，ORR为38.6%，中位PFS 5.7个月。

基于一项Ⅰ期/Ⅱ期临床试验AF-001JP中对未经ALK抑制剂治疗的ALK阳性患者93.5%的ORR，2014年Alectinib首先在日本获得批准^[27]^。而2015年Alectinib被FDA批准用于克唑替尼耐药的ALK阳性晚期NSCLC患者是基于两项Ⅱ期临床试验^[23]^：NP28671和NP28673。前者入组87例克唑替尼耐药患者，ORR为48%，中位PFS 8.1个月^[28]^；后者入组138例克唑替尼耐药患者，ORR为50%，DCR为69%，中位PFS 8.9个月^[29]^。

### 2.2 尚未获批的ALK-TKIs

Brigatinib（AP26113）同时是ALK、ROS1和EGFR抑制剂。一项Ⅰ期/Ⅱ期临床试验2016年报告的结果中，Brigatinib治疗克唑替尼耐药和未经克唑替尼治疗的患者ORR分别为72%（51/71）和100%（8/8），整体人群的中位PFS达到13.2个月^[30]^。同年报告的Ⅱ期临床研究ALTA入组克唑替尼耐药患者222例，A组接受Brigatinib每日90 mg，B组接受每日90 mg共7 d后增量到每日180 mg，ORR分别为46%和54%，中位PFS分别为8.8个月和11.1个月^[31]^。Lorlatinib（PF-06463922）也是一种多靶点药物，它是ALK和ROS1抑制剂。目前正在进行Ⅰ期/Ⅱ期临床试验，2016年ASCO报告其Ⅰ期研究阶段的最新结果：入组41例ALK阳性患者及12例ROS1阳性患者（均大部分经过TKIs治疗），ORR为50%^[31]^。其他在早期临床研究中的新一代ALK-TKIs还包括X-396、ASP3026、TSR-011、Entrectinib、CEP-28122等。

综上，新一代ALK-TKIs在克唑替尼获得性耐药的患者中已展现了不俗的疗效。同时已经可以初步看出，这些药物在未经克唑替尼治疗的ALK阳性患者中疗效更好。2016年ASCO报告了Alectinib一项Ⅲ期临床试验初步结果：Alectinib对比克唑替尼用于未经ALK-TKIs治疗过的ALK阳性患者，截至报告，Alectinib组中位PFS尚未达到（95%CI: 20.3-无法评估），而克唑替尼组中位PFS 10.2个月^[32]^。2016年WCLC报告了Ceritinib对比克唑替尼一线治疗ALK阳性患者的Ⅲ期临床试验ASCEND-4，该研究达到了其首要终点：Ceritinib组PFS较克唑替尼组显著延长（16.6个月*vs* 8.1个月；HR=0.55，95%CI：0.42-0.73；*P* < 0.000, 01），ORR也明显提高（72.5% *vs* 26.7%）^[33]^。因此，新一代ALK-TKIs的角色同样并非限制在克唑替尼获得性耐药的解救治疗。同时研究证实，各种ALK-TKI对于不同的继发性突变敏感性不同（表 2），甚至有Lorlatinib耐药后回头应用克唑替尼重新有效的报道^[34]^。因此，未来理想的方式应该是动态监测耐药患者突变状态的变化，并选择相应的ALK-TKI。

**2:** 各种ALK-TKIs对ALK激酶域不同继发性突变位点的敏感性差异 Sensitivities of different ALK-TKIs to different secondary ALK kinase domain mutations

Mutations	Crizotinib	Ceritinib	Alectinib	Brigatinib	Lorlatinib
1151Tins	Resistant^[68]^	Resistant^[68]^	Sensitive^[69]^	Sensitive^[70]^	NR
C1156Y	Resistant^[68]^	Resistant^[68]^	Sensitive^[69]^	Sensitive^[70]^	Sensitive^[71]^
D1203N	Resistant^[72]^	NR	NR	Sensitive^[70]^	NR
E1210K	Sensitive^[73]^	Resistant^[73]^	Resistant^[73]^	Sensitive^[70]^	Sensitive^[73]^
F1174C	Resistant^[68]^	Resistant^[68]^	NR	Sensitive^[70]^	NR
F1174L	Resistant^[74]^	NR	Sensitive^[71]^	Sensitive^[71]^	Sensitive^[71]^
F1174V	Resistant^[68]^	Resistant^[68]^	Sensitive^[75]^	Sensitive^[70]^	NR
F1245C	Resistant^[76]^	Sensitive^[76]^	NR	NR	NR
G1123S	NR	Resistant^[77]^	Sensitive^[77]^	NR	NR
G1202R	Resistant^[68]^	Resistant^[68]^	Resistant^[69]^	Sensitive^[70]^	Sensitive^[78]^
G1269A	Resistant^[68]^	Sensitive^[68]^	Sensitive^[71]^	Sensitive^[71]^	Sensitive^[71]^
I1171N	Resistant^[73]^	Resistant^[73]^	Resistant^[75]^	Sensitive^[70]^	Sensitive^[73]^
I1171S	NR	NR	Resistant^[75]^	NR	NR
I1171T	Resistant^[68]^	Sensitive^[68]^	Resistant^[75]^	Sensitive^[68]^	Sensitive^[71]^
L1152R	Resistant^[68]^	Resistant^[68]^	Sensitive^[69]^	Sensitive^[70]^	NR
L1196M	Resistant^[68]^	Sensitive^[68]^	Sensitive^[75]^	Sensitive^[71]^	Sensitive^[71]^
L1198F	Sensitive^[34]^	Resistant^[34]^	Resistant^[34]^	Sensitive^[70]^	Resistant^[34]^
S1206Y	Resistant^[68]^	Sensitive^[68]^	NR	Sensitive^[70]^	Sensitive^[71]^
V1180L	NR	Sensitive^[79]^	Resistant^[80]^	Sensitive^[70]^	Sensitive^[80]^
NR: not reported.					

## 3 新治疗靶点的拓展

在中国，*EGFR*突变及*EML4-ALK*融合基因分别约占NSCLC患者的约41%^[35]^和8.65%^[36]^。这意味着除此之外，还有约一半的患者无法从靶向治疗中获益。因此，除了EGFR及ALK靶点的纵深研究，寻找新的治疗靶点也是当务之急。

### 3.1 KRAS

*KRAS*基因突变是高加索裔NSCLC患者最常见的驱动基因突变，发生率约30%^[37-39]^，亚裔人群中约占5.8%^[40]^。*KRAS*突变在肺癌中的发现已有30年之久^[41]^，但以其作为治疗靶点的临床研究却屡屡失败。唯独近年来针对KRAS下游通路的MEK抑制剂在*KRAS*突变患者中展现出一定的临床前景。2012年MEK抑制剂司美替尼报告了一项Ⅱ期临床研究结果^[42]^，共入组87例*KRAS*突变的Ⅲb期/ Ⅳ期NSCLC患者，随机接受多西他赛联合司美替尼或多西他赛联合安慰剂，结果显示司美替尼组较安慰剂组ORR和PFS均有显著优势，ORR分别为37%和0（*P* < 0.000, 1），中位PFS分别为5.3个月和2.1个月（HR=0.58, 90%CI: 0.42-0.79; *P*=0.014），OS也有延长趋势，中位OS分别为9.4个月和5.2个月（HR=0.80; 90%CI: 0.56-1.14; *P*=0.21）。然而遗憾的是近期阿斯利康公司宣布司美替尼用于*KRAS*突变型NSCLC的Ⅲ期临床试验^[43]^以失败告终，希望将来最终试验结果的发表能阐明失败的原因，给以后*KRAS*突变型肺癌的研究以启示。

### 3.2 ROS1

*ROS1*基因编码一种胰岛素受体家族的酪氨酸激酶受体，*ROS1*基因重排可导致其所编码受体的酪氨酸激酶活性异常，导致下游多条致癌信号通路的激活。*ROS1*基因重排在我国患者中的阳性率与高加索裔无差异，约为1%-2%^[44-46]^。克唑替尼同时是ALK、ROS1和MET抑制剂，其Ⅰ期临床试验PROFILE 1001中包含一个ROS1的扩展队列，共入组50例*ROS1*基因重排的NSCLC患者，ORR为72%，中位治疗持续时间17.6个月，中位PFS为19.2个月^[47]^。从而克唑替尼被FDA批准用于*ROS1*基因重排的NSCLC患者。2016年ASCO报告了一项克唑替尼治疗*ROS1*基因重排的东亚患者的Ⅱ期临床试验，共入组129例ROS1阳性、ALK阴性、全身治疗≤3线的进展期NSCLC患者，克唑替尼客观缓解率达到69%，且与治疗线数无关，DCR为84%，中位治疗持续时间7.8个月，中位PFS尚未达到^[48]^。

### 3.3 MET

*MET*基因是肝细胞生长因子受体蛋白（hepatocyte growth factor receptor, HGFR）的编码基因^[49]^。MET信号通路的异常激活可由多种形式导致，如基因突变、基因扩增、蛋白的过表达等^[50, 51]^。它的异常激活，可以作为原发驱动基因，也是EGFR-TKIs获得性耐药的重要机制之一^[49]^。克唑替尼Ⅰ期临床试验PROFILE 1001的MET扩增队列中，克唑替尼单药显示出一定的临床疗效，但ORR仅33%^[52]^。2016年ASCO报告Capmatinib（INC280）的Ⅰ期临床数据，在免疫组化3+的患者中ORR为29%，基因拷贝数≥5的患者中ORR为63%^[53]^。最值得期待的疗效预测因子可能是*MET*基因14外显子跳跃突变：2015年，Paik等^[54]^报告小分子MET抑制剂（克唑替尼、卡博替尼）在MET 14外显子跳跃突变的NSCLC患者中可能疗效卓越。2016年ASCO会议中，PROFILE 1001研究报告了MET 14外显子跳跃突变队列应用克唑替尼治疗的初步结果，截至报告为止，15例可评价疗效的患者中10例患者达到PR^[55]^。从以上结果看来，*MET*基因的扩增、过表达及突变似乎均可作为MET抑制剂的疗效预测因子，其中以14外显子突变的疗效预测意义最为显著。基因扩增、过表达及突变作为疗效预测因子是否具有内在逻辑的相关性值得进一步探索。

### 3.4 RET

*RET*基因重排是一种已知在甲状腺癌中可作为治疗靶点的驱动基因，它在NSCLC的发生率约1.5%^[56]^。目前NSCLC中*RET*基因的相关临床研究多是在评价已在甲状腺癌中获批的RET抑制剂治疗RET重排NSCLC的疗效。如2015年ASCO报道卡博替尼在一项单臂的Ⅱ期临床研究中治疗20例*RET*重排NSCLC的结果，在18例可评价疗效的患者中，ORR为28%，中位PFS 7个月（95%CI：3个月-未达到），中位OS未达到^[57]^。2016年ASCO报告凡德他尼治疗RET重排NSCLC的两项Ⅱ期单臂临床试验：一项来自日本，入组19例患者，可评价疗效者17例，ORR为53%，中位PFS 4.7个月^[58]^；另一项来自韩国，共入组18例患者，ORR 17%^[59]^。

### 3.5 BRAF

*BRAF*也是一种在其他瘤种中可作为治疗靶点的驱动基因：*BRAF* V600E突变见于约40%-60%的恶性黑色素瘤患者^[60]^，这些患者接受BRAF抑制剂达拉菲尼、Vemurafenib治疗疗效突出。在肺腺癌中*BRAF*突变的发生率约1%-5%，其中大部分为V600E突变^[61]^。在一项Vemurafenib的Ⅱ期篮子试验中入组了19例BRAF V600E阳性的NSCLC患者，ORR为42%，中位PFS 7.3个月^[62]^；达拉菲尼治疗BRAF V600E阳性NSCLC的Ⅱ期临床试验入组84例患者，ORR 33%，中位PFS 5.5个月^[63]^。另一项Ⅱ期临床研究重复了恶性黑色素瘤的成功模式，将达拉菲尼与MEK抑制剂曲美替尼联合用于治疗BRAF V600E阳性NSCLC，57例患者，ORR为63%，中位PFS 9.7个月^[64]^。

### 2.6 HER2

HER2与EGFR同属于ERBB受体家族。在乳腺癌中，HER2扩增发生率约20%，且可以作为抗HER2单抗或TKIs的疗效预测因子^[65]^。在NSCLC中，虽然HER2扩增有约2%-4%的发生率，但以其作为靶点进行的临床研究均告失败。而在肺腺癌中，有约1%-2%的患者存在HER2 20外显子突变^[66]^。目前尚缺乏前瞻性临床研究数据。但2012年有报告使用阿法替尼治疗了3例HER2 20外显子突变型NSCLC，3例患者均达到部分缓解（partial response, PR）^[67]^。另一项欧洲的回顾性队列研究显示，这部分患者接受HER2抑制剂（阿帕替尼等TKI单药或曲妥珠单抗联合化疗）的治疗可达到50%的ORR和83%的疾病控制率，中位PFS为5.1个月^[66]^。

## 4 小结

综上所述，目前根据驱动基因的不同，我们至少可以将NSCLC分为具有治疗指导意义的7种分子亚型：*EGFR*突变型、*EML4-ALK*基因融合型、*ROS-1*基因融合型、*CMET*高表达/扩增/突变型、*RET*基因融合型、*BRAF*突变型、*HER-2*突变型。第三代EGFR-TKIs和新一代ALK-TKIs的出现解决了一代药物的耐药问题，使EGFR和ALK阳性患者的生存进一步延长，而针对其他的少见靶点，也或多或少有临床证据支持相应的药物应用。同时近年来基因检测技术的快速发展使得我们能更合理地利用这些药物进行治疗决策：如NGS能利用较少的标本一次进行多种基因的检测、液体活检技术使我们可以动态检测耐药患者的基因状态改变。因此，晚期非小细胞肺癌靶向治疗的临床应用将由此带来深度和广度上的进一步革新。然而，新的问题总是接踵而来：我们能否打破药物敏感、耐药、新药研发、再耐药的无尽循环？部分少见靶点的疗效差强人意（RET、BRAF、HER-2），如何解决？能否进一步扩展靶向治疗的版图（KRAS等）？期待未来的研究可以使这些问题得以解决。
